# The Application of Extension in Fractures by Means of Adhesive Plaster

**Published:** 1850-12

**Authors:** 


*The Application of Extension in Fractures by means of Adhesive Plaster.*

[Allusion was made in the August number of the Journal to the use of adhesive plaster as a means of applying extension in fractures. Understanding that Dr. Josiah Crosby's method was not stated with perfect accuracy, a note was addressed asking for information upon the point. The following is a portion of the reply. Ed.]

My first plan was to apply two strips of fresh spread English Adhesive Plaster, one on either side of the leg, wide enough to cover at least half the diameter of the limb from above the knee to the malleolar processes, below which the straps were left floating a foot or more without plaster. A circular strap was then applied above the knee—above the calf of the leg, and another above the malleolar processes, and over these the roller bandage; but in the last case I applied the longitudinal straps from below the knee to the ankle, relying on the roller bandage without the circular straps. This is undoubtedly preferable, as the circular straps might be injurious in case the limb should become much swollen.

This was a case where considerable extension was necessary, and was continued until union had taken place, without the slightest alteration in the first dressing. On the 32d day, the roller, which was applied from the toes to above the knee, was removed in presence of several physicians, and the straps were adhering well to the limb, and no appearance of excoriation of the skin, and the patient had never uttered a complaint from the extension. His answer to the inquiry, "what the sensation was" would be, "It feels as if my leg was in the mud, and I was trying to pull it out."

I am now treating a compound oblique fracture of the tibia about three inches above the ankle joint, (the fibula also is broken,) making both extension and counter extension in a most perfect manner with the adhesive straps, and without the slightest inconvenience to the patient—no complaint from the dressings, whatever. The counter extension is made from *below* the knee, and the extension from two inches of the lower part of the leg and the foot.

I have treated two cases of fracture of the clavicle in children of two years old, with nothing but adhesive straps, with as good success as I ever had with the old methods, and not half the trouble.

Your ob't serv't,

JOSIAH CROSBY.

[*From the New Hampshire Journal of Medicine.*]

